# Current evidence of flexible ureteroscopic laser lithotripsy under local anesthesia: feasibility, safety, and clinical application

**DOI:** 10.3389/fmed.2026.1942070

**Published:** 2026-10-14

**Authors:** Hongbo Chen, Shiyu Xing, Shenglong Shi, Shufan Lai

**Affiliations:** 1 Department of Urology, The Third Affiliated Hospital of Gannan Medical University, Ganzhou, Jiangxi, China; 2 Department of Anesthesiology, The Third Affiliated Hospital of Gannan Medical University, Ganzhou, Jiangxi, China

**Keywords:** feasibility, flexible ureteroscopic laser lithotripsy, flexible ureteroscopy, local anesthesia, safety, urolithiasis

## Abstract

Flexible ureteroscopic laser lithotripsy (FURL) is commonly performed under general or neuraxial anesthesia, but local anesthesia (LA) has been explored as an alternative for selected patients. This structured narrative review summarizes current evidence for LA-FURL while explicitly separating LA alone, LA combined with systemic analgesia or conscious/moderate sedation, peripheral regional blocks, and neuraxial anesthesia. An updated literature search identified a small but growing body of direct LA-FURL evidence, consisting predominantly of retrospective or single-arm clinical series together with recent larger prospective cohorts. These studies suggest that FURL can be completed and tolerated under LA in selected patients, with stone-free rates generally in the range reported by individual series; however, differences in stone burden, anesthesia protocols, access-sheath technology, imaging modality, timing, and definitions of stone-free status preclude a pooled estimate or a claim of equivalence to general anesthesia. Observational complication profiles are encouraging, but comparative safety and non-inferiority versus general anesthesia have not been established. Technical measures such as careful patient preparation, gentle instrumentation, suction-assisted access sheaths, attention to intrarenal pressure, and selective pre-stenting may be useful, but most have not been validated as LA-FURL-specific standards. Evidence from ureteroscopy under conscious sedation is considered separately because it represents a distinct anesthetic strategy. Overall, LA-FURL is a promising option for appropriately selected patients and experienced teams, particularly when avoidance of general or neuraxial anesthesia is desirable, but the current evidence base remains heterogeneous and largely nonrandomized. Prospective comparative studies with standardized definitions of procedural completion, stone-free status, pain, complications, patient-reported outcomes, and cost are required before broader recommendations can be made.

## 1 Introduction

Flexible ureteroscopic laser lithotripsy (FURL) is a minimally invasive endoscopic treatment for renal and proximal ureteral stones. In this review, FURL refers specifically to flexible ureteroscopy or flexible ureterorenoscopy in which laser lithotripsy is performed within the upper urinary tract. Semirigid ureteroscopic laser lithotripsy (URSL), which is primarily used for ureteral stones and has different technical and anesthetic considerations, is outside the core scope of this review. The term retrograde intrarenal surgery (RIRS) is retained only when describing the terminology used in individual source studies (1).

General anesthesia (GA) and neuraxial anesthesia remain commonly used for FURL because they provide immobility and reliable analgesia. Interest in LA-FURL has developed mainly from the need to reduce anesthetic exposure in selected high-risk patients and to explore less resource-intensive ambulatory pathways. Direct clinical evidence now includes series of flexible ureterorenoscopy performed with local anesthetic alone or predominantly local anesthetic techniques, as well as studies using LA with additional systemic analgesia or light sedation (2–5). These approaches should not be interpreted as interchangeable with GA, neuraxial anesthesia, or conscious sedation.

The available LA-FURL literature is heterogeneous. Studies differ in patient selection, stone burden, access-sheath technology, local anesthetic regimen, use of systemic analgesics or sedatives, and outcome definitions. Accordingly, claims regarding feasibility, safety, stone-free rate (SFR), recovery, and cost must be linked to the anesthesia modality and procedural setting actually studied rather than extrapolated across distinct techniques.

Potential practical advantages of LA-FURL include avoidance of GA or neuraxial anesthesia in selected patients, preservation of spontaneous ventilation, and the possibility of simplified perioperative pathways. However, these potential advantages should not be assumed to translate into superior recovery, lower cost, or equivalent safety without direct comparative evidence. Patient cooperation, pain tolerance, stone complexity, surgeon experience, and the ability to convert anesthesia when necessary remain central considerations.

This review therefore focuses on the current clinical evidence for FURL performed under LA, with particular attention to the directness of the evidence. Evidence from LA combined with systemic sedation/analgesia is analyzed separately, while studies of peripheral regional blocks, neuraxial anesthesia, semirigid URSL, conventional FURL under other anesthesia modalities, and non-urological procedures are used only as contextual or indirect evidence where clearly identified.

## 2 Methods

### 2.1 Review design and scope

This article is a structured narrative review rather than a systematic review or meta-analysis. The primary question was whether FURL for renal or proximal ureteral stones can be performed under LA with acceptable procedural feasibility, patient tolerance, and short-term clinical outcomes. The review does not treat topical/intraluminal LA, LA with systemic sedation or analgesia, peripheral regional blocks, neuraxial anesthesia, and GA as equivalent anesthetic exposures.

### 2.2 Search strategy

PubMed/MEDLINE and the Cochrane Library were searched from database inception through 4 September 2026. The PubMed search combined the following concepts: (“flexible ureteroscopy” OR “flexible ureterorenoscopy” OR FURL OR “retrograde intrarenal surgery”) AND (“local anesthesia” OR “local anaesthesia” OR “topical anesthesia” OR “conscious sedation”) AND (“laser lithotripsy” OR urolithiasis OR “urinary stone” OR “renal stone”). The same concepts were adapted for the Cochrane Library. Reference lists of relevant clinical reports and reviews were also screened for additional studies.

### 2.3 Eligibility and evidence classification

Direct evidence was defined as human clinical evidence involving flexible upper-tract ureteroscopy with laser lithotripsy performed under a local anesthetic strategy. Studies using systemic analgesia or conscious/moderate sedation in addition to LA were retained but classified separately. Semirigid URSL was excluded from the core evidence synthesis; mixed URS series were used only when flexible/intrarenal outcomes were separately reported or when the limitation was explicitly stated. Peripheral regional blocks, spinal/epidural anesthesia, GA comparisons that did not include LA, and non-urological studies were considered indirect evidence and were not used to establish LA-FURL-specific efficacy or safety.

### 2.4 Study selection and appraisal

Potentially relevant reports were screened by title and abstract and then reviewed at full-text level when needed to determine procedural and anesthetic relevance. Because this was a narrative review, no pooled effect estimate was calculated and no formal risk-of-bias tool was applied. Evidence was qualitatively appraised according to study design, sample size, directness to LA-FURL, presence of a comparator, definition of the anesthesia protocol, procedural completion or conversion, SFR definition and follow-up, pain assessment, complications, and discharge outcomes. Conclusions give greatest weight to direct LA-FURL evidence and identify adjacent or extrapolated evidence explicitly.

## 3 Background and evolution of local anesthesia in urology

### 3.1 Classification of local anesthesia techniques

For this review, anesthesia modalities are classified according to the source of analgesia and the degree of systemic sedation. This distinction is essential because the available evidence does not support combining all non-GA techniques into a single “local anesthesia” category.

LA alone or predominantly local anesthesia refers to topical or intraluminal local anesthetic applied to the urethra or urinary tract while the patient remains awake and without neuraxial or general anesthesia. Direct upper-tract experience includes flexible ureterorenoscopy performed with local anesthetic lubricating gel and FURL series using topical urethral local anesthetic as the principal local technique (3, 4). The principal clinical studies informing LA-FURL and adjacent upper-tract ureteroscopy under local anesthesia or conscious sedation are summarized in Table 1.

**Table 1:** Principal clinical studies informing LA-FURL and adjacent upper-tract ureteroscopy under local anesthesia or conscious sedation.

Study/evidence class	Population and stone characteristics	Anesthesia category and protocol	Completion/conversion	SFR definition and result	Pain/tolerability	Complications/discharge
Pai et al. 2019 (3) Direct LA flexible ureterorenoscopy; retrospective, single center	78 patients considered unsuitable for GA/regional anesthesia; median stone size 8 mm; upper-tract urolithiasis	LA alone/predominantly LA: local anesthetic lubricating gel per urethra; no neuraxial or GA	All patients tolerated the procedure; none abandoned because of pain	82% after one procedure; residual-fragment/imaging threshold not specified in the PubMed abstract	Tolerability reported; no standardized numeric pain value in the abstract	No complication > Clavien grade 2; median length of stay 1 day
Zhang et al. 2023 (4) Direct LA-FURL; retrospective	56 patients, 66 procedures; unilateral mean stone size 20.24 ± 5.45 mm; bilateral total burden 29.40 ± 3.89 mm	LA + systemic premedication: pethidine 1–2 mg/kg IM + promethazine 25 mg 30 min preop; 10 mL oxybuprocaine urethral gel (30 mg)	All procedures completed without conversion to GA/regional anesthesia; no additional analgesia	No remaining stone on KUB and/or NCCT; day-30 SFR 85.1% unilateral and 85.0% bilateral renal units	Intraop VAS 3.83 ± 0.53 unilateral; 3.10 ± 0.74 bilateral	Complications 8.7% unilateral and 10.0% bilateral; postoperative stay 3.43 ± 1.70 and 3.20 ± 1.40 days
Peng et al. 2023 (2) Direct LA flexible ureteroscopy; large single-center clinical series	614 patients with urinary stones treated from 2021 to 2023	LA protocol reported in the article; abstract does not provide sufficient detail to classify all systemic adjuncts	Procedural completion/conversion not uniformly reported in the abstract	Overall SFR 83.4%; definition/follow-up threshold not specified in the abstract	Highest VAS scores occurred during sheath insertion; anxiety scores decreased during/after surgery	18 postoperative fevers; 12 ureteral injuries; no severe complication reported; LA cost about $40 in the local DRG framework
Jain et al. 2022 (6) Adjacent evidence: conscious-sedation ureteroscopy, not LA alone; retrospective	99 URS; 73 for urolithiasis; 42 intrarenal and 23 proximal ureter procedures	Urologist-directed conscious sedation; median 3 mg midazolam and 100 μg fentanyl	83.8% overall success; 81.0% intrarenal; 70.0% proximal ureter	Stone-case SFR 80.8%; definition not specified in the abstract	Patient tolerability data were a stated study limitation	No intraoperative complications or hospital admissions reported
Chang et al. 2025 (11) Direct LA-FURL; retrospective comparative device study	372 patients with 2–4 cm renal stones; FANS *n* = 187 vs traditional UAS *n* = 185	Both groups underwent FURL under LA; detailed LA protocol not provided in the abstract	Completion/conversion not reported in the abstract	One-time SFR 78.6% FANS vs 57.8% traditional UAS	Pain outcome not reported in the abstract	Postoperative fever 2.1% vs 8.1%; steinstrasse 1.1% vs 5.9%
Zhang et al. 2026 (5) Direct LA upper-tract ureteroscopy; multicenter single-arm prospective study	395 patients with stones ≤30 mm and persistent asymptomatic bacteriuria/pyuria after antibiotic therapy	FANS-UAS-assisted upper-tract ureteroscopy under LA; detailed drug regimen not provided in the abstract	Conversion not the primary reported endpoint	Immediate stone-free rate 80.5%; final SFR was a secondary endpoint	Transient intraoperative VAS elevation with reduction after surgery	Postoperative fever 2.8%; study focused on high infectious-risk patients
Tang et al. 2026 (13) Adjacent/mixed evidence: LA-URS; retrospective	151 patients: flexible RIRS *n* = 82 and rigid URS *n* = 69	LA with a standardized multimodal perioperative protocol including VAS-guided rescue analgesia	98.0% overall success for the mixed cohort; flexible subgroup success not separately reported	SFR not a primary result in the PubMed abstract	Peak pain during mechanical dilation (VAS 3.68 ± 1.52); lithotripsy VAS 2.03 ± 1.53	No major complications reported

FURL, flexible ureteroscopic laser lithotripsy; LA, local anesthesia; GA, general anesthesia; UAS, ureteral access sheath; FANS, flexible and navigable suction ureteral access sheath; SFR, stone-free rate; VAS, visual analog scale; KUB, kidney-ureter-bladder radiography; NCCT, non-contrast computed tomography. The table separates direct LA-FURL evidence from adjacent conscious-sedation or mixed-URS evidence; values should not be pooled because definitions and populations differ.

LA with systemic analgesia or conscious/moderate sedation is a separate strategy. Some FURL protocols combine topical LA with preoperative opioids or sedating medication, and conscious-sedation ureteroscopy commonly uses agents such as midazolam and fentanyl (4, 6). Outcomes from these studies may inform patient tolerance but should not be attributed to LA alone.

Peripheral nerve blocks, including quadratus lumborum or sacral plexus blocks, are regional anesthesia techniques rather than local mucosal anesthesia. Neuraxial anesthesia, including spinal, epidural, or combined spinal-epidural anesthesia, is also distinct. Studies comparing regional or neuraxial anesthesia with GA are therefore treated as contextual anesthesia evidence rather than direct evidence for LA-FURL (7–9).

Throughout the remainder of this review, the term LA-FURL is reserved for flexible ureteroscopic laser lithotripsy performed with a local anesthetic strategy. When systemic sedation/analgesia is added, this is stated explicitly. This framework prevents evidence from semirigid URSL, regional blocks, neuraxial anesthesia, or conscious sedation from being interpreted as equivalent LA-FURL evidence.

### 3.2 Motivation for the transition from general anesthesia to local anesthesia

The rationale for investigating LA-FURL is primarily clinical and logistical rather than evidence of proven superiority over GA. GA provides reliable immobility and analgesia but may be undesirable in selected patients with substantial anesthetic risk or when rapid recovery and limited anesthesia resources are important considerations.

Direct clinical series illustrate this rationale. Pai et al. reported local-anesthetic flexible ureterorenoscopy in patients considered unsuitable for GA or regional anesthesia, while Peng et al. described a large institutional LA flexible-ureteroscopy experience motivated in part by resource and cost considerations (2, 3). These reports support investigation of LA-FURL as an alternative pathway but do not constitute randomized evidence that LA is safer or more effective than GA.

Ambulatory and office-based endourological practice also provides a practical motivation for minimizing anesthetic intensity. Prospective office-based data suggest that selected endoscopic urological procedures can be performed under topical anesthesia with acceptable patient tolerance, but the ureteroscopy subgroup is small and the procedures are heterogeneous (10). These findings are therefore contextual rather than definitive evidence for routine LA-FURL.

Resource utilization is another consideration. Avoiding GA or neuraxial anesthesia may reduce the need for anesthesia recovery resources in selected settings, but reported costs are highly dependent on local reimbursement systems, procedural setting, and the specific LA protocol. Cost observations from individual LA-FURL cohorts should not be generalized as universal savings.

Accordingly, this section is limited to why LA-FURL is being explored. Comparative outcomes such as procedural completion, complications, SFR, recovery, and patient-reported outcomes are evaluated separately below using the most direct evidence available.

LA-FURL is therefore being investigated as an additional option for carefully selected patients and settings in which avoidance of GA or neuraxial anesthesia is desirable. Whether it offers comparable efficacy, safety, or cost-effectiveness remains an open comparative question.

## 4 Patient selection and preoperative assessment

### 4.1 Indications and contraindications

Patient selection is central to LA-FURL, but the available literature does not establish a single validated stone-size threshold. Direct studies include patients with relatively small stones as well as cohorts with substantially larger renal stone burdens. Pai et al. reported a median stone size of 8 mm, Zhang et al. treated unilateral and bilateral upper-tract stones with mean burdens around 20–30 mm, and a recent LA-FURL cohort specifically evaluated renal stones measuring 2–4 cm (3, 4, 11). These differences indicate that stone size should be considered a pragmatic marker of anticipated procedural complexity rather than an evidence-based absolute cutoff for LA-FURL.

Likewise, no direct evidence identified in this review supports an estimated glomerular filtration rate below 30 mL/min as an absolute contraindication to LA-FURL. Patients with impaired renal function or high anesthetic risk have been included in local-anesthetic ureterorenoscopy series (3, 4). Practical reasons to avoid an awake LA procedure include allergy to the intended local anesthetic, inability to cooperate or remain safely positioned, uncontrolled pain or anxiety despite planned rescue measures, or inability to provide timely conversion to deeper anesthesia if needed.

Large or complex stone burden, difficult anatomy, bilateral disease, anticipated prolonged instrumentation, active uncontrolled infection, and prior access difficulty should be treated as factors that may reduce tolerability or increase procedural complexity rather than universal LA-specific contraindications. Their importance depends on operator experience, device availability, drainage strategy, and the ability to stage the procedure.

Pregnancy and other special populations require individualized anesthetic assessment. Ureteroscopy under LA with light sedation has been described during pregnancy, but this represents a distinct, highly selected strategy and does not establish routine LA-FURL indications for pregnant patients (12).

Preoperative imaging remains necessary to define stone location, burden, anatomy, obstruction, and potential access difficulties. For LA-FURL specifically, imaging is best used to estimate procedural complexity and the likelihood that the planned operation can be completed while maintaining patient tolerance, rather than to apply unvalidated universal thresholds.

### 4.2 Patient psychological preparation and cooperation

Successful LA-FURL depends on informed patient cooperation because the patient remains responsive to discomfort and procedural manipulation. Preoperative counseling should explain expected sensations, the possibility of additional analgesia or sedation, and the circumstances in which the procedure would be staged or converted. Direct LA flexible-ureteroscopy experience also suggests that anxiety changes during the perioperative period and that sheath insertion or ureteral dilation can be important pain-generating steps (2, 13).

No validated LA-FURL-specific maximum operative time was identified. A 60-minute threshold has been proposed from experience in other procedural settings, but direct LA-FURL evidence does not validate this as a universal cutoff. Procedure duration is better interpreted in relation to patient tolerance and stone complexity. In a second-look flexible nephroscopy series, procedures lasting more than 30 min were associated with higher pain scores, whereas a direct LA-FURL series reported successful bilateral procedures with mean operative times around 90 min (4, 14). These observations do not justify a universal time cutoff.

Pre-stenting is similarly better viewed as a pragmatic access strategy rather than an established requirement for LA-FURL. In the Zhang et al. series, most patients were pre-stented and the authors favored this approach to facilitate access-sheath placement; however, the study did not randomize pre-stenting and therefore cannot establish that 1–2 weeks of pre-stenting improves LA-specific completion or tolerance (4). Pre-stenting may be considered when it is otherwise clinically or technically indicated.

When systemic analgesia or conscious/moderate sedation is added, the anesthetic strategy should be described separately from LA alone. The choice and titration of systemic agents should be individualized according to comorbidity, monitoring resources, and the need to preserve spontaneous ventilation and patient responsiveness; evidence from non-urological procedures should not be presented as an LA-FURL-specific drug protocol.

Psychological preparation, realistic expectations, gentle technique, and a predefined rescue or conversion plan are reasonable components of LA-FURL practice. Operative-time limits, mandatory pre-stenting intervals, and specific sedation regimens should not be presented as established standards until supported by direct prospective evidence.

## 5 Local anesthesia techniques: drug selection and administration protocols

### 5.1 Commonly used local anesthetics and their concentrations

Local anesthetics differ in onset, duration, and systemic toxicity, but general pharmacological properties should be distinguished from evidence supporting a particular drug for LA-FURL. The current direct clinical literature does not establish a preferred local anesthetic agent, concentration, or maximum procedure-specific dose for FURL.

Direct flexible-ureteroscopy reports have used topical urethral local anesthetic as part of the anesthetic strategy. Pai et al. described flexible ureterorenoscopy using local anesthetic lubricating gel per urethra, while Zhang et al. used oxybuprocaine urethral gel together with preoperative pethidine and promethazine (3, 4). These protocols demonstrate feasibility in their respective cohorts but should not be interpreted as evidence that one local anesthetic regimen is superior.

Lidocaine, ropivacaine, and bupivacaine have well-described general pharmacological differences, including differences in cardiotoxicity and duration of action. However, much of the evidence cited for specific concentrations or comparative toxicity comes from non-urological, laboratory, dental, or regional-anesthesia settings (15–17). Such evidence is relevant to drug safety but does not establish LA-FURL-specific dosing recommendations.

Direct LA-FURL comparative evidence is insufficient to recommend against bupivacaine specifically for ureteral irrigation or to define another agent as superior on this basis. Any use of local anesthetic within the urinary tract should instead account for cumulative dose, route of administration, systemic absorption, patient comorbidity, and the possibility of local-anesthetic systemic toxicity.

Likewise, combinations with epinephrine and buffering with bicarbonate are pharmacological techniques described in other clinical settings (18, 19). They should not be presented as routine LA-FURL strategies without direct evidence of benefit and safety in ureteroscopic practice.

Topical local anesthesia is a recurring component of reported LA-FURL protocols, but current evidence is insufficient to recommend a single agent, concentration, volume, or adjunct as the standard regimen. Protocols should be described exactly as studied and interpreted within their clinical context.

### 5.2 Drug administration methods and timing

Drug administration in LA-FURL should be described according to the route actually used in the supporting study. Direct reports most commonly describe topical urethral local anesthetic gel, sometimes combined with systemic analgesia or light sedation (3, 4). Direct LA-FURL evidence does not support describing renal-pelvic instillation of 10–20 mL of anesthetic 5–10 min before FURL as the most commonly employed or standardized administration method.

Local-anesthetic instillation into the renal collecting system has been reported in other upper-tract procedures, including second-look flexible nephroscopy after PCNL, but this is anatomically and procedurally different from retrograde FURL and should be considered adjacent evidence rather than a standard LA-FURL administration protocol (14).

Irrigation-system pressure and measured intrarenal pressure are distinct variables and should not be used interchangeably. Specific values such as 30 mmHg for irrigation-system pressure and 40 cmH₂O for intrarenal pressure arise from different measurement contexts and are not interchangeable or validated as universal LA-FURL targets. Direct LA-FURL studies support the general principle of avoiding excessive intrarenal pressure, particularly when pain or infectious risk is a concern, but they do not establish a single mandatory pressure threshold (4, 5, 11, 13).

If additional local anesthetic is required intraoperatively, it should be delivered through an appropriate catheter or endoscopic working channel according to the chosen protocol; a guidewire is not a drug-delivery conduit. Any supplementation should account for cumulative local-anesthetic dose and patient monitoring.

Current evidence supports accurate reporting of the studied route and careful control of irrigation and intrarenal pressure rather than a universal LA-FURL drug volume, timing, or pressure target.

## 6 Key points of surgical procedures and equipment selection

### 6.1 Considerations for flexible ureteroscopes and lithotripsy instruments

Instrument selection and efficient stone treatment are particularly relevant when FURL is performed in an awake or lightly sedated patient, but most evidence concerning ureteroscope caliber, laser settings, and fragmentation strategy comes from FURL performed under conventional anesthesia. These data should therefore be regarded as general FURL principles unless an LA-specific comparison is available.

Smaller-caliber flexible ureteroscopes and atraumatic handling may plausibly reduce mechanical discomfort, yet published comparisons of scope design have not been conducted specifically to test pain or completion under LA (20, 21). Their relevance to LA-FURL is therefore extrapolative.

Low-energy, higher-frequency laser settings and dusting techniques are widely used in contemporary FURL. A direct LA-FURL series using a suctioning access sheath employed low-energy/high-frequency dusting settings, demonstrating that this technique can be used successfully under LA (4). However, the study did not randomize laser settings; therefore, specific energy-frequency ranges should not be presented as proven to improve LA tolerance (4, 22–24).

Similarly, dusting may reduce repeated basket manipulation, but evidence comparing dusting with fragmentation and basket retrieval is primarily based on general FURL outcomes rather than LA-specific pain or conversion endpoints (22, 24). The choice should remain individualized according to stone characteristics and the surgeon's strategy.

Gentle scope manipulation, avoidance of excessive torque, and appropriate use of an access sheath are reasonable technical principles. Suction-enabled access sheaths have been used in several recent LA-FURL cohorts and may facilitate drainage and fragment evacuation, but their observed benefits cannot be separated completely from patient selection, surgeon experience, and other aspects of the protocol (4, 5, 11).

Technical measures that shorten unnecessary manipulation and preserve visualization are likely helpful in LA-FURL, but specific scope sizes, laser settings, and stone-fragmentation strategies should be described as study protocols or extrapolated FURL principles rather than established LA-FURL standards.

### 6.2 Irrigation and drainage management

Irrigation management is important during FURL because elevated intrarenal pressure may contribute to pain, pyelovenous backflow, and infectious complications. However, pump or irrigation-system pressure is not equivalent to intrarenal pressure measured within the collecting system, and the literature does not define a universal LA-FURL-specific pressure threshold (25, 26).

Recent LA-FURL studies have emphasized low-pressure technique and suction-assisted drainage. In the Zhang et al. series, a suctioning ureteral access sheath was used in all patients, and later LA cohorts have evaluated flexible and navigable suction access sheaths in larger patient populations (4, 5, 11). These studies support the feasibility of pressure-conscious drainage strategies, but they do not isolate an optimal intrarenal pressure target.

The use of warmed irrigation fluid has been proposed to reduce ureteral spasm and discomfort, but the supporting evidence is not specific enough to establish warmed irrigation as an LA-FURL standard. It should be described as a potentially useful extrapolated strategy rather than a proven requirement.

Likewise, lidocaine-containing irrigation solutions have theoretical appeal for local analgesia, but direct LA-FURL evidence is insufficient to conclude that they reduce additional analgesic requirements. Any intraluminal anesthetic irrigation should be considered investigational or protocol-specific until dose, absorption, and clinical benefit are better characterized.

Current evidence supports avoiding excessive intrarenal pressure, maintaining adequate drainage and visualization, and reporting the irrigation or suction system used. Exact pressure, temperature, and anesthetic-irrigation protocols require prospective LA-FURL-specific validation.

## 7 Intraoperative pain management and patient monitoring

### 7.1 Pain assessment tools and management strategies

Pain assessment is essential during LA-FURL because patient-reported discomfort can guide pauses, additional analgesia, staging, or conversion. VAS or numeric rating scales are commonly used, but the specific thresholds reported in individual studies should not be interpreted as validated LA-FURL standards.

Direct LA-FURL series demonstrate that pain can remain acceptable in selected patients, although the most painful procedural step varies. In Zhang et al., mean intraoperative VAS scores were 3.83 ± 0.53 for unilateral procedures and 3.10 ± 0.74 for bilateral procedures, while Peng et al. reported that the highest pain scores occurred during access-sheath insertion (2, 4). These findings support real-time pain monitoring but do not define a universal cutoff for continuation or conversion.

A recent mixed LA-URS series using a standardized perioperative protocol reported peak pain during ureteral mechanical dilation and lower pain during lithotripsy, with an overall procedural success rate of 98%; however, the cohort included both flexible and rigid ureteroscopy and the flexible subgroup success rate was not separately reported (13). This evidence is relevant to pain mechanisms but cannot be treated as a pure LA-FURL outcome.

VAS thresholds such as ≤4 for acceptable pain or >6 as a trigger to pause have been used in individual protocols, but they have not been validated as universal LA-FURL standards. Clinical decisions should also consider pain trajectory, anxiety, vital signs, procedural stage, and the availability of rescue analgesia or anesthesia conversion.

Preoperative anxiety can influence procedural tolerance. Counseling and communication are therefore reasonable components of an LA-FURL pathway, but the relationship between anxiety scores and LA-FURL completion requires prospective validation.

For future studies, pain should be reported at standardized procedural steps, together with the need for rescue medication, sedation, staging, or conversion. This would allow pain thresholds to be evaluated rather than assumed.

### 7.2 Adjunctive use of sedative agents

Systemic sedation should be considered a distinct anesthetic strategy rather than an extension of LA alone. LA-FURL with adjunctive analgesia or light sedation may be clinically useful, but its feasibility and safety cannot be attributed solely to the local anesthetic component.

Ureteroscopy under urologist-directed conscious sedation provides relevant adjacent evidence. In the study by Jain et al., 99 ureteroscopies were performed under conscious sedation; the overall procedural success rate was 83.8%, with success rates of 81.0% for intrarenal and 70.0% for proximal ureteral procedures. The median sedation requirement was 3 mg midazolam and 100 μg fentanyl (6). Because this was a conscious-sedation cohort rather than LA alone, these rates are not used as LA-FURL completion estimates.

Some direct FURL reports also used systemic premedication together with topical local anesthetic. For example, Zhang et al. administered pethidine and promethazine before topical oxybuprocaine (4). Such protocols are more accurately described as LA with systemic analgesic/sedative adjuncts rather than local anesthesia alone.

Comparisons among dexmedetomidine, midazolam, propofol, and other sedatives in non-urological procedures can inform general sedation pharmacology but do not establish an LA-FURL-specific preferred agent (27–29). Conscious or moderate sedation should therefore not be described as a standard of care for LA-FURL in the absence of procedure-specific guideline support.

Reports of LA alone and LA plus systemic sedation/analgesia should be analyzed separately, with explicit documentation of agents, doses, monitoring, rescue medication, and conversion.

## 8 Analysis of existing evidence on feasibility and safety

### 8.1 Feasibility: procedural completion

Procedural completion or conversion is the most direct measure of feasibility and should not be conflated with SFR or success after secondary procedures. Available LA-FURL studies use heterogeneous feasibility endpoints, so a pooled completion range cannot be justified from the current evidence.

In one retrospective series of 78 patients undergoing local-anesthetic flexible ureterorenoscopy, all patients tolerated the procedure and none was abandoned because of pain (3). In the Zhang et al. LA-FURL cohort, all 56 patients (66 procedures) were completed without conversion to GA or regional anesthesia and without additional intraoperative analgesia (4). These studies provide direct evidence of procedural tolerability in selected populations.

Other large LA-FURL studies primarily report SFR, operative time, complications, or pain rather than a uniform completion endpoint. Peng et al. reported outcomes in 614 patients undergoing flexible ureteroscopy under LA, and Chang et al. reported 372 LA-FURL procedures for 2–4 cm renal stones, but these reports should not be converted into procedural-completion percentages unless completion or conversion is explicitly defined (2, 11).

A recent multicenter single-arm study of FANS-UAS-assisted upper-tract ureteroscopy under LA further supports feasibility in a selected high-infectious-risk population, but its primary outcomes were stone clearance and postoperative fever rather than a comparative anesthesia endpoint (5). Likewise, the 98% success rate reported by Tang et al. applies to a mixed cohort of 82 flexible and 69 rigid LA ureteroscopies and should not be treated as a pure LA-FURL rate (13).

We did not identify a meta-analysis that supported the previously stated pooled LA-FURL procedural-completion estimate of 89.7%. Feasibility is therefore summarized using study-specific completion or conversion data rather than a pooled estimate.

Technological developments such as suctioning access sheaths and improved flexible ureteroscopes may facilitate efficient procedures, but observational device studies cannot establish that the technology itself makes LA equivalent to GA or guarantees completion.

Direct series indicate that LA-FURL can be completed in carefully selected patients, but definitions and reporting are inconsistent. At present, a single pooled completion rate is not justified, and future studies should report planned procedure completion, conversion to deeper anesthesia, staging, and abandonment separately.

### 8.2 Safety evaluation indicators

Safety data for LA-FURL are derived mainly from observational cohorts without concurrent GA controls. The available direct evidence suggests an acceptable short-term complication profile in selected patients, but it does not establish equivalence or non-inferiority to GA.

In the 614-patient Peng et al. series, 18 patients developed postoperative fever and 12 experienced ureteral injury, with no severe complication reported (2). In the Zhang et al. LA-FURL series, complications occurred in 8.7% of unilateral and 10.0% of bilateral patients, including fever, stent pain, urosepsis requiring additional antibiotics, and steinstrasse (4). Pai et al. reported no complications above Clavien grade 2 (3).

Recent LA-FURL cohorts using suction-enabled access sheaths provide additional safety observations. Chang et al. reported lower fever and steinstrasse rates with FANS than with a traditional access sheath in patients with 2–4 cm stones, while a multicenter study in patients at high infectious risk reported postoperative fever in 2.8% of 395 patients (5, 11). These comparisons evaluate devices or selected populations under LA, not LA versus GA.

These observational data do not establish that LA complication rates are equivalent or non-inferior to those under GA. Absence of severe events in selected cohorts cannot establish anesthesia equivalence without an appropriate comparative design and prespecified margin.

Local-anesthetic systemic toxicity should remain a clinical safety consideration whenever topical or intraluminal local anesthetic is used. The absence of reported severe toxicity in the available LA-FURL series is reassuring but should not be interpreted as proof that the risk is negligible (30, 31).

## 9 Clinical outcomes and limits of comparative evidence

### 9.1 Stone-free rate (SFR)

SFR should be interpreted using the definition, imaging modality, timing, stone burden, and whether the value represents a single procedure or a final result after further intervention. Direct evidence does not support fixed LA-versus-GA SFR or recurrence figures across these heterogeneous studies; study-specific LA-FURL estimates are therefore more appropriate. Pai et al. reported an SFR of 82% after one procedure, although the abstract does not specify a uniform imaging threshold (3). Zhang et al. defined stone-free status as no residual stone on KUB radiography and/or non-contrast CT and reported day-30 SFRs of 85.1% for unilateral procedures and 85.0% for bilateral renal units (4). Peng et al. reported an overall SFR of 83.4% in 614 LA flexible-ureteroscopy patients, but the definition and follow-up threshold are not specified in the abstract (2). In patients with 2–4 cm renal stones, Chang et al. reported one-time SFRs of 78.6% with FANS and 57.8% with a traditional access sheath; this was a device comparison in which both groups underwent LA-FURL, not an LA-versus-GA comparison (11). A recent multicenter single-arm LA study in a high-infectious-risk population reported an immediate stone-free rate of 80.5% (5). These values demonstrate that clinically useful stone clearance is achievable in selected LA-FURL cohorts, but they are not directly comparable and cannot establish equivalence to GA. Future reports should specify imaging modality, residual-fragment threshold, time point, and whether SFR refers to the index procedure or final clearance.

### 9.2 Postoperative recovery and patient satisfaction

Evidence regarding postoperative recovery, cost, analgesic use, and patient satisfaction after LA-FURL remains limited. No direct source identified in this review supports universal LA-versus-GA figures for discharge time, percentage cost savings, satisfaction scores, or postoperative analgesic use; such outcomes should therefore be reported only when directly measured in comparable groups.

Direct LA flexible-ureteroscopy studies report outcomes within their own institutional pathways. Pai et al. reported a median length of stay of 1 day (3). Zhang et al. reported mean postoperative hospitalization of 3.43 ± 1.70 days after unilateral LA-FURL and 3.20 ± 1.40 days after bilateral procedures (4). Peng et al. reported low anesthesia-related cost within the Chinese DRG framework, but this was not a randomized LA-versus-GA economic comparison (2).

Prospective office-based endourology data suggest that topical anesthesia can reduce facility costs and that many patients prefer the office setting, but only a small subgroup underwent ureteroscopy and the study included heterogeneous procedures (10). These findings are supportive of ambulatory feasibility but should not be generalized as LA-FURL-specific comparative cost-effectiveness.

Evidence from dexmedetomidine-versus-propofol comparisons under combined spinal-epidural anesthesia addresses a different anesthetic strategy and should not be used to infer LA-versus-GA recovery or satisfaction outcomes (27).

Patient satisfaction under LA-FURL is clinically important but is not measured consistently across the direct literature. Pain scores, willingness to repeat the procedure, rescue medication, time to mobilization, discharge criteria, and validated satisfaction instruments should be reported prospectively.

At present, LA-FURL may permit streamlined recovery in selected pathways, but claims of shorter hospitalization, lower overall cost, lower analgesic requirements, or superior satisfaction relative to GA require direct prospective comparative studies.

## 10 Implementation challenges and optimization strategies

### 10.1 Technical training and experience

LA-FURL demands a high level of technical proficiency, particularly in flexible ureteroscope manipulation, to ensure stable stone retrieval even when patients exhibit slight movements during the procedure. Mastery of soft ureteroscope handling is essential because the absence of GA or deep sedation means that patient cooperation and involuntary motions can challenge the surgeon's ability to maintain precise control of the endoscope and laser lithotripsy instruments. This requirement underscores the importance of surgeons having advanced skills in flexible ureteroscopy, including navigation within the renal collecting system, stone fragmentation, and fragment extraction, all while minimizing patient discomfort and procedural complications.

Given these challenges, early LA-FURL experience is best developed under supervision by surgeons with substantial endourological expertise. Initial cases may reasonably be selected for lower anticipated procedural complexity, but the available evidence does not validate a universal stone-size or calyceal-location threshold for training cases. Case selection should instead consider stone burden and location, anticipated access difficulty, patient cooperation, prior ureteral access, and the availability of rescue or conversion pathways. General FURL literature supports the importance of operator experience, but these data should not be interpreted as an LA-FURL-specific numerical selection rule (25, 32).

Simulation-based training is emerging as a valuable adjunct to traditional apprenticeship models in FURL skill acquisition. High-fidelity virtual reality (VR) simulators and 3D-printed anatomical models of the upper urinary tract have been developed and externally validated to replicate the tactile and visual feedback of flexible ureteroscopy and laser lithotripsy. For instance, the “Tor Vergata” 3D printed models have demonstrated face, content, and construct validity, showing significant differences in operative times and technical skill scores between novices and experts. Repeated simulation sessions lead to measurable improvements in surgical performance metrics, suggesting that simulation training can accelerate the learning curve and enhance operative efficiency and safety before live patient procedures (33). The integration of VR and physical models into standardized curricula may be particularly beneficial for training surgeons in LA-FURL, where patient movement and limited anesthesia depth add complexity.

Institution-specific operating procedures can support consistent LA-FURL practice, particularly by defining the local anesthetic strategy actually used, monitoring requirements, rescue analgesia or sedation, criteria for staging or conversion, and emergency access to anesthesiology support. However, specific drug doses, pain-score trigger points, and conversion thresholds should be presented as protocol-specific unless prospectively validated. Such pathways are most appropriately used for local quality assurance and training rather than presented as universal LA-FURL standards.

Technical efficiency is relevant to patient tolerance, but specific laser settings, dusting strategies, and fragment-retrieval approaches are supported mainly by general FURL literature rather than LA-specific comparative trials. Ureteral access sheaths can facilitate instrument exchange and drainage, while preoperative alpha-blocker therapy such as silodosin has been studied for facilitating access-sheath placement in flexible ureterorenoscopy (34). These measures may be useful in selected LA-FURL pathways, but they should be described as general or extrapolated technical strategies rather than established methods for improving LA-FURL completion or stone-free rates.

Technical expertise remains important for LA-FURL because patient movement, discomfort, and the need for real-time communication may add procedural complexity. Supervised clinical experience, simulation-based training, and institution-specific rescue and conversion protocols are reasonable components of implementation, but their effect on LA-FURL-specific safety or efficacy has not yet been established in comparative studies.

### 10.2 Patient selection algorithms and decision-making tools

Validated patient-selection tools for LA-FURL are not currently available. Candidate selection is therefore based on clinical judgment integrating stone burden and location, anticipated access difficulty and operative time, comorbidity, prior stenting or ureteral access, anxiety, communication, and the patient's willingness to undergo an awake or lightly sedated procedure.

No derivation or validation study was identified for an “LA-FURL Smoothness Score” or for a numerical threshold defining candidacy. Variables such as stone burden, access difficulty, anxiety, and anticipated operative time may inform future prediction models, but formal derivation and external validation would be required before clinical use.

Similarly, no validated artificial-intelligence model was identified that predicts suitability specifically for LA-FURL or selects an anesthesia modality for FURL. Machine-learning methods may eventually integrate imaging, patient characteristics, anxiety measures, and procedural history, but this remains a research concept rather than established clinical decision support.

Institutional day-surgery pathways for LA-FURL may also be explored, but they should be developed around explicit inclusion criteria, rescue analgesia and conversion capability, post-procedure observation, and discharge criteria. Current observational data are insufficient to define a universal pathway.

Direct LA-FURL studies support the concept that patient selection materially influences feasibility, but they do not validate a single score or algorithm (2–5). Future prospective cohorts should derive and externally validate prediction models using standardized completion, pain, conversion, and complication endpoints.

## 11 Future research directions and clinical prospects

### 11.1 Novel local anesthetics and carriers

Long-acting local-anesthetic formulations and drug-delivery systems are potential future strategies, but they have not been clinically established for LA-FURL. Liposomal or gel-based local-anesthetic platforms have demonstrated prolonged delivery in experimental or non-urological settings, including ropivacaine formulations, but this evidence does not demonstrate ureteral efficacy, safety, or a 4–6 h clinical anesthetic effect during FURL (35, 36).

Physical methods intended to enhance local-drug penetration, such as iontophoresis or ultrasound-assisted delivery, are likewise conceptual in the context of the urinary tract. Their tissue effects, urothelial safety, systemic absorption, and practical role during retrograde endoscopy require preclinical evaluation before clinical LA-FURL testing.

Biodegradable or drug-eluting ureteral devices could theoretically provide prolonged local analgesia after ureteroscopy, but the concept remains experimental. No LA-FURL clinical evidence identified in this review supports routine use of local-anesthetic-coated biodegradable stents.

These technologies should therefore be presented as future research opportunities rather than current advances in LA-FURL care. Development should proceed through pharmacokinetic, toxicology, dose-finding, and prospective clinical studies before claims regarding improved tolerability or reduced systemic analgesia are made.

### 11.2 Need for multicenter comparative studies

Current direct evidence for LA-FURL remains dominated by retrospective cohorts and single-arm clinical series, although larger recent cohorts and multicenter prospective data have expanded the evidence base (2–5). These studies establish clinical feasibility in selected populations but do not provide high-quality comparative evidence against GA or neuraxial anesthesia.

Future comparative studies should use standardized primary endpoints. Procedural completion should be defined independently of SFR and should include conversion to deeper anesthesia, procedure abandonment, and planned staging. SFR should specify imaging modality, residual-fragment threshold, time point, and whether the outcome follows the index procedure or additional intervention. Pain should be recorded at predefined procedural steps, and complications should use a standardized classification.

Stratified analyses should examine stone burden and location, sex, age, comorbidity, prior stenting, anxiety, anticipated operative time, access-sheath technology, and surgeon experience. These variables may influence both selection for LA and the probability of successful completion and therefore are important potential confounders.

Health-economic evaluation should be embedded within prospective comparative studies rather than inferred from institutional charges alone. Relevant outcomes include anesthesia staffing and recovery resources, procedure duration, hospital stay, unplanned admission, conversion, repeat procedures, complications, and patient time costs.

Multicenter studies are particularly important because current LA-FURL practice is concentrated in experienced centers using different local anesthetic, analgesic, access-sheath, and perioperative protocols. External validation is necessary before one institutional pathway is generalized.

High-quality prospective comparative studies, ideally randomized when ethically and practically feasible, are therefore required to determine which patients benefit from LA-FURL and whether safety, efficacy, patient experience, and cost-effectiveness are comparable with established anesthesia strategies.

### 11.3 Individualized anesthesia strategies and monitoring

Individualized anesthesia may combine local anesthetic with carefully titrated systemic analgesia or sedation when clinically appropriate. This should be viewed as a spectrum of distinct anesthetic strategies rather than a single “multimodal comfort surgery” standard. The intensity of systemic medication should be documented because it materially affects interpretation of patient tolerance and respiratory risk.

Urological evidence supports the feasibility of conscious-sedation ureteroscopy in selected patients, and light sedation has also been described for ureteroscopy during pregnancy (6, 12). These reports suggest a role for tailored sedation but do not validate non-pharmacological adjuncts such as music or virtual-reality distraction specifically for LA-FURL.

EEG- or bispectral-index-guided sedation can assist assessment of sedation depth in other procedural settings, but an EEG-based automatic pain-feedback system that directly adjusts local-anesthetic delivery has not been validated for LA-FURL. Closed-loop analgesia should therefore be presented as an experimental concept rather than a current clinical capability (28, 37).

Future research could evaluate whether standardized communication, distraction techniques, physiological monitoring, and titrated systemic adjuncts improve LA-FURL completion or patient-reported experience. Such studies should distinguish the contribution of local anesthesia from that of systemic sedation and other co-interventions.

Ethical safeguards remain essential. Patient preference, capacity to cooperate, availability of rescue analgesia or anesthesia conversion, and the absence of economic pressure to avoid appropriate anesthesia should be explicit components of any LA-FURL pathway.

Accordingly, individualized and technology-assisted anesthesia for FURL is a promising research direction, but most proposed components remain unvalidated. Their use should be tested prospectively before they are described as established improvements in LA-FURL care.

## 12 Conclusion

The current literature suggests that FURL under local anesthesia may be a feasible option for carefully selected patients when performed by experienced teams with an appropriate rescue or conversion pathway. Direct studies report successful completion or acceptable tolerance in selected cohorts and clinically useful stone clearance, but the evidence base remains heterogeneous and is dominated by observational or single-arm designs. Published SFRs cannot be compared directly without accounting for stone burden, imaging modality, follow-up, residual-fragment thresholds, and repeat procedures. Likewise, available safety data are encouraging but do not establish equivalence or non-inferiority to general anesthesia. Evidence for shorter hospitalization, lower cost, reduced postoperative medication use, or greater patient satisfaction is limited and setting-dependent. Local anesthesia alone should be distinguished from LA combined with systemic analgesia or conscious/moderate sedation, and regional or neuraxial anesthesia should not be grouped with LA. Technical strategies such as pre-stenting, suction-enabled access sheaths, pressure-conscious irrigation, and specific laser settings may facilitate treatment in some series, but most have not been validated as LA-FURL-specific standards. Future multicenter prospective comparative studies should use standardized definitions of procedural completion, conversion, SFR, pain, complications, patient-reported outcomes, and cost. Until such evidence is available, LA-FURL is best regarded as a promising, selectively applicable approach rather than an established equivalent alternative to GA.
